# Applying the Modified Health Belief Model (HBM) to Korean Medical Tourism

**DOI:** 10.3390/ijerph17103646

**Published:** 2020-05-22

**Authors:** Hyun-Jeong Ban, Hak-Seon Kim

**Affiliations:** School of Hospitality and Tourism Management, Kyungsung University, 309 Suyoungro, Nam-Gu, Busan 48434, Korea; helenaban@ks.ac.kr

**Keywords:** visiting intention, Korean medical tourism, health belief model (HBM)

## Abstract

The purpose of this study is to investigate how foreigners’ health beliefs influence their visiting intentions to Korean medical tourism. This study used an online survey engine (docs.google.com/forms) to collect data from foreigners who are from India, Nepal, Bangladesh, Vietnam, and Mongolia, who are potential Korean medical tourists. Out of 213 questionnaires collected, 200 questionnaires (93.9%) were used for the statistical analysis. The Confirmatory factor analysis (CFA) revealed that six dimensions, “Experience”, “Susceptibility”, “Severity”, “Barrier”, “Benefit”, and “Visiting Intention”, had construct validity; Cronbach’s alpha coefficient was tested for item reliability. It is expected that four variables in the health belief model (HBM) that are determined by objective and logical thinking processes will affect the choice of Korean medical tourism. The results showed that Susceptibility, Severity, Barrier, and Benefit had significant effects on Visiting Intention and was a valid measurement to determine “Visiting Intention”.

## 1. Introduction

Medical tourism has been growing rapidly worldwide, from about USD 10 billion in 2012 to about USD 33 billion in 2019. Along with this growth, various countries are striving to become competitive as medical tourism destinations. Medical tourism is about traveling, culture, and recreation activities in nearby tourist attractions while receiving medical services such as treatments, health examinations, and beauty treatments [1]. Medical tourists visiting Korea are mostly from China, the United States, Japan, Russia, and Kazakhstan. In addition, they were found to be affected by a variety of factors, including high medical costs, long waiting times, and low medical technology levels [2]. According to the prior study, medical tourism has two major characteristics. One is that medical tourism makes tourists journey longer, and the other one is that the cost of tourism is more expensive than general tourism [3]. According to Beladi, Chao, Ee, and Hollas, many countries have been actively promoting medical tourism to stimulate economic growth [4]. Therefore, many countries and local governments are interested in medical tourism, which is classified as a high value-added industry.

Korean medical technology has unsurpassed competency in treatments for difficult diseases, such as cancer, cardiovascular and spinal disorders, and complicated procedures such as transplants. In addition, hospitals in Korea boast of excellent medical practitioners and facilities such as digit subtraction angiography units, gamma camera, mammography, MRI, PET, and CT [5]. Recently, Korean medicine is globally recognized as next-generation medicine, along with Complementary Alternative Medicine (CAM). In terms of severe diseases such as cancer, musculoskeletal disorders, and intractable diseases, the scientific treatment methods and cases of Korean medicine have already been proved and are constantly in research [6,7,8]. The Korean government has strict regulations over the hospitals to ensure optimum medical services. The 5-years survival rate of cancer patients in Korea is the highest compared to the rate in other advanced countries such as the United States, Canada, and Japan [9]. In addition, Korea has a competitive physical environment, such as the number of beds per population and the number of CTs and MRIs held at the level of advanced countries. Based on the price competitiveness of dental implants, gastrointestinal treatments, gastric bypass, breast prosthesis, and Lasik procedures, the medical service company achieved remarkable growth in a short period of time despite being a latecomer to medical tourism. Therefore, Korea is establishing itself as one of the major destinations for medical tourism [10].

Many studies are being done on the development of medical tourism. Fetscherin and Stephano [11] published a paper presenting the Medical Tourism Index, a new type of country-based performance measure to assess the attractiveness of a country as a medical tourist destination. They found out “Country Environment”, “Tourism Destination”, “Medical Tourism Cost”, and “Medical Facility and Service” are significant with the Medical Tourism Index. Saragih and Jonathan [12] examined Indonesian consumers through the use of behavioral lenses to examine their medical tourism experience in Malaysia. The Indonesians are willing to have medical treatment in Malaysia, and resources and capabilities are the essential factors when choosing medical tourism. Hallem and Barth [13] focused on the impact of the Internet and presented a conceptualization of international, social, and relational Internet functions for understanding medical tourist behavior. Kim, Arcodia, and Kim [14] discovered that Korean medical tourism has been facilitated by the effect of advanced Korean brand power, and more importantly, tourism activities for companions and extra support for patients’ convenience are identified as important success factors of Korean medical tourism. However, although medical tourism is a new concept by convergence, research on patients’ behavior in terms of medical tourism is insufficient. If the Korean tourism association is interested in promoting medical tourism, they need to understand the patient’s perception of disease and its effect on behavior. There is a Health Belief Model (HBM) that is widely used in the medical community and conceptualizes patients’ health-related behaviors. Therefore, HBM makes their behavior predictable, and generally includes activities to recover from unhealthy conditions as well as activities to maintain and promote health [15].

This model was developed in the 1950s by social psychologists working in the US Public Health Services [16]. In the early stages of development, it was developed for the purpose of explaining and predicting specific actions. Later, it was also found useful in explaining health-related behaviors. The theory suggests that the incidence of certain diseases can be controlled by identifying significant factors in the performance of human health behaviors. The core of HBM is how individuals perceive the various environments surrounding health and how they influence individual behavior. Individuals, in particular, have the most advantageous behavior, considering the costs and benefits of choosing alternatives [17]. The HBM consists of four sub-components: perceived Susceptibility, where individuals believe they are susceptible to a particular disease; perceived Severity that individuals believe may be potentially serious in their health; perceived Benefits that are perceived to benefit from taking preventive action in reducing the severity or susceptibility of a particular disease; finally, there are perceived Barriers to taking preventive action rather than the benefits of taking a preventive action [18].

Visiting Intention is defined as the thought or plan to visit. Intent means the probability that a person’s expected or planned future action will be shifted to an action [19]. Therefore, the visiting intention is an important concept in predicting the behavior of tourists, and the purpose of using HBM is to analyze foreigners’ perception of cancer and Korean medical service to determine their visiting intentions to Korea. Korea is already well known for medical services in beauty and plastic surgery; there are many prior studies [20,21,22,23,24]. Therefore, this study seeks to find out the intention of visiting Korea to use medical services for cancer, based on HBM.

## 2. Literature Review

### 2.1. Medical Tourism Experience in Korea

The quality of Korean healthcare has been ranked as being among the world’s best. It had the OECD’s highest colorectal cancer survival rate at 72.8%, significantly ahead of Denmark’s 55.5% or the UK’s 54.5%. It ranked second in cervical cancer survival rate at 76.8%, significantly ahead of Germany’s 64.5% or the US at 62.2%. Hemorrhagic stroke 30-day in-hospital mortality per 100 hospital discharges was the OECD’s third-lowest at 13.7 deaths, which was almost twice as low as the US at 22.3 or France’s 24 deaths. For ischemic stroke, it ranked second at 3.4 deaths, which was almost a third of Australia’s 9.4 or Canada’s 9.7 deaths. South Korean hospitals ranked fourth for MRI units per capita and sixth for CT scanners per capita in the OECD. It also had the OECD’s second-largest number of hospital beds per 1000 people at 9.56 beds, which was over triple that of Sweden’s 2.71, Canada’s 2.75, the UK’s 2.95, or the US at 3.05 beds. Moreover, cancer treatment in South Korea has brought positive results for international patients. South Korea posts the highest 5-year survival rates for stomach, thyroid, and prostate cancer. It also tops the charts on breast cancer treatment. Many hospitals have unique resources that go from state-of-the-art facilities such as proton-therapy, robotic surgery, to highly specialized centers. Additionally, merging modern techniques with Korean traditional medicine to boost the level of treatment success [25].

In general, the medical industry can be referred to as the medical service industry, the pharmaceutical and medical supplies industry, and the medical information industry, all of which can be seen as economic activities that supply goods or services to protect, maintain, and enhance human health. The US and Japan have already focused on the health and medical industries since the 1980s. However, the government, industry, and academia have limited the medical industry to its function as a health and medical service supplied to the domestic market. It has also been perceived as a subject of regulation and control and neglected in terms of industrial policy. The law on Korean medical tourism has been eased; the number of Korean medical tourists increased from 60,201 in 2009 to 378,967 in 2018 [26]. Korean medical tourism is led by the government, and the government is active in preparing institutional devices to revitalize medical tourism. In fact, in addition to providing institutional support for attracting foreign patients through the revision of the Medical Service Act in 2009, the Korean government announced that it would select global healthcare as a new growth engine project, along with the green technology industry and IT convergence industry in 2009, and actively participate in the promotion of medical tourism. The Ministry of Health and Welfare prepares conditions for hospitals to accept overseas patients in the field of medical services, while the Ministry of Culture, Sports and Tourism and the Korea Tourism Organization are in charge of overseas promotion marketing for medical tourism. The Ministry of Foreign Affairs and Trade and the Ministry of Justice are in charge of medical tourism visas. As such, the medical tourism support system is strategically carried out through the sharing of roles among government ministries [5].

**Figure 1:**
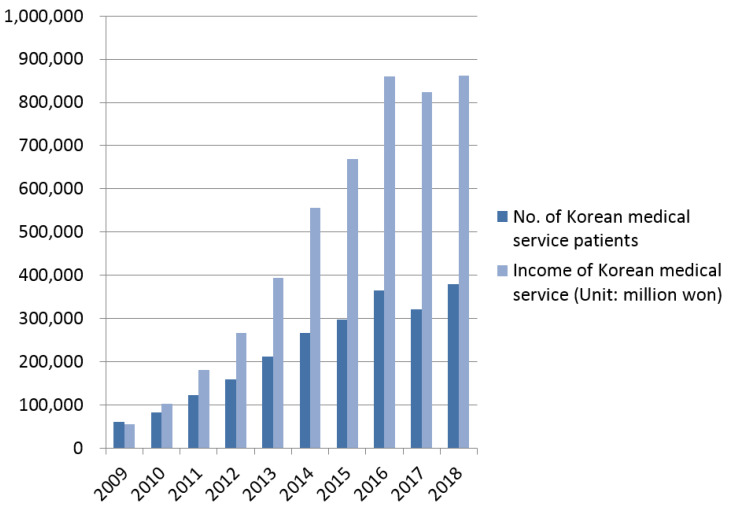
Growth of Korean medical tourism.

As a result of the government’s active support policies, Korea is becoming a major medical tourism country in Asia despite being a latecomer in the medical tourism industry. As shown in Figure 1, the number and income of patients using Korean medical tourism continued to increase until 2016, and the growth rate in 2017 decreased. Korean medical technology competitiveness ranks among the top OECD member countries. Health care costs are also one-third of those in the United States and two-thirds of those in Japan, with strong price competitiveness. In the early days of medical tourism, medical tourists visited Korea in the order of the US (23.0%), Japan (21.6%), and China (7.8%), but in 2018, the medical tourists were mainly from China (35.0%), the US (13.4%), and Japan (7.3%). In particular, the proportion of medical tourists from the US and Japan decreased, while that of China (35.0%), Russia (7.0%), and Kazakhstan (4.1%) increased [27].

Nevertheless, compared to the existing Asian leading medical tourism countries, Korean medical tourism lacks recognition and suffers from a narrow market size. In addition, countries adjacent to Korea are actively seeking to attract medical tourists. In the case of China, it is creating a “Shanghai International Medical Zone” by attracting world-class medical institutions such as Harvard Medical School in the US to China, and in the case of Japan, it is seeking to foster the medical industry as a next-generation growth engine as part of “Abenomics”. Therefore, fierce competition is expected with Korea in the future. In the case of Korea, it currently has a 4.3% share in the Asian medical tourism market and less than 1% of the global medical tourism market. Therefore, it has yet to gain a large share in the medical tourism industry. As Korea has just started medical tourism, it seems necessary to develop potential medical tourists for the continued growth trend [27,28].

### 2.2. Theory of HBM (Health Belief Model)

HBM was developed by social psychologists (Hochbaum, Keeles, Leventhal, and Rosenstock) in the early 1950s. The model was developed to understand people’s practice of preventing diseases or not performing early checkups on diseases. There is a field of positive and negative values in the space of human life, in which disease is an area of negative value, taking a phenomenological approach that what actions a person will take when he or she wants to escape from a disease is not determined by the physical environment but by the subjective perception of the person [16,17]. In addition, HBM is a representative model that explains people’s health behaviors, and the variables that make up HBM have been studied as the main factors predicting domestic and foreign health behavior practices, such as obesity, high blood pressure, AIDS, smoking, and various cancers [18]. However, health behaviors vary depending on the disease, and HBM’s variables explain health behaviors, suggesting different significance and outcome values for each study.

**Figure 2:**
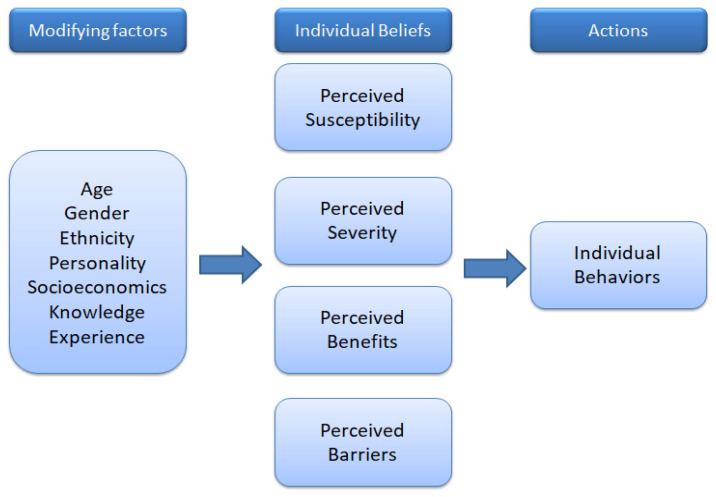
Health belief model (HBM).

Nevertheless, HBM is one of the most commonly used models in health-related studies, explaining and predicting human health practices as well as health-preventive behavior. HBM assumes that individuals take preventive action when they feel they are more likely to develop a disease (“perceived susceptibility”), and that there are severe negative effects (“perceived severity”) of the disease and “benefits” from adopting health behaviors, and that there are fewer “barriers” when taking health actions, as shown in Figure 2 [29]. Modifying factors (left column) affect these perceptions, as do individual behaviors (right column). HBM is based on an expectancy-value framework and assumes that health decision-making is a rational process. According to Larsen [30], the illness experience is the foundation of understanding individual and behavior. In addition, beliefs are the ultimate psychological determinants of behavior, and behavioral beliefs are assumed to influence attitudes [31]. In the current study, related variables in HBM are integrated to better understand tourists’ visiting intentions and travel satisfaction. However, few studies have been conducted to test the impacts of health beliefs on visiting intention in the tourism context.

Some researchers indicate that the perceived threat positively correlates with healthy behavior, and the perception of threats would encourage people to act to reduce their risk [32]. In the tourism context, if the perceived susceptibility of potential risks is higher, the more people will demand medical services in technologically advanced environments [33]. Therefore, in this study, the hypothesis of applying modified HBM was established based on the existing HBM. The first part of the hypothesis is to learn about the impact of potential customer’s experience on the four factors (“Susceptibility”, “Severity”, “Benefit”, and “Barrier”) of HBM. In the second part of the hypothesis, the four factors of HBM will explore potential customers’ visiting intention to Korean medical tourism.

## 3. Materials and Methods

In order to address this study’s objective, quantitative methods were employed. A structured survey questionnaire was used to investigate several factors: (a) Disease Experience; (b) Susceptibility, Severity, Benefit, and Barrier; (c) Visiting Intention to Korean medical tourism; (d) demographic information. For questionnaire validity and reliability, the Confirmatory Factor Analysis (CFA), and Cronbach’s alpha test were employed. Therefore, the following hypotheses were established on a theoretical basis:

**Hypotheses** **1-1.** *Potential medical tourists with disease experience will have a high level of susceptibility.*

**Hypotheses** **1-2.** *Potential medical tourists with disease experience will have a high level of severity.*

**Hypotheses** **1-3.** *Potential medical tourists with disease experience will have a high level of benefit.*

**Hypotheses** **1-4.** *Potential medical tourists with disease experience will have a high level of barrier.*

**Hypotheses** **2-1.** *Potential medical tourists with a high level of susceptibility will have a high level of visiting intention to Korean medical tourism.*

**Hypotheses** **2-2.** *Potential medical tourists with a high level of severity will have a high level of visiting intention to Korean medical tourism.*

**Hypotheses** **2-3.** *Potential medical tourists with a high level of benefits will have a high level of visiting intention to Korean medical tourism.*

**Hypotheses** **2-4.** *Potential medical tourists with a low level of barriers will have a high level of visiting intention to Korean medical tourism.*

The questions in the survey are focused on Korean medical services for cancer treatment, as shown in Table 1. The questionnaire of the HBM contained four parts. The HBM is a comprehensive questionnaire, which helps to predict a patient’s behavior to cure the diseases. Besides, the basis of this model is encouraging the participants to take part in and increasing their awareness of health beliefs, which creates acceptable behavior. Feeling the threatening risk of cancer (susceptibility) is the first step for preventive action. Afterwards, the intensity and life-threatening complications of the medical tourism (severity), believing in accuracy and the benefits of the preventive programs (benefits), and the inhibitory factors of accurate behavior, which have less importance than its advantages (barrier), and, finally, performing therapeutic behavior [34].

The questions in the survey were taken from a prior study of medical tourism with HBM, and the validity of the questionnaire was proven by the previous study [35,36,37]. The study used a convenience online sampling method. As it targets potential medical tourists living in many other countries, such as India, Nepal, Bangladesh, Vietnam, and Mongolia, who do not use Korean medical tourism, an online questionnaire was used. Questionnaires using an online survey engine (docs.google.com/forms) were distributed from 1–28 February 2020. A graphical representation of the specified model for this study can be seen in Figure 1. It is a modification of the Rosenstock, Strecher, and Becker studies on social learning and the HBM [18]. This model incorporates the objective experience as a predictor for medical tourism in the process of health belief and visiting intentions for respondents. The survey was conducted on non-Korean foreigners. The questionnaire was conducted in English, and 45 questionnaires were completed by translating English into Mongolian with the help of experts. Then, 213 copies were collected after distributing online questionnaires, and only 200 copies were used after 13 unsatisfactory responses, which were not completed or had the same answer for every question, were discarded. SPSS 25.0 and AMOS 20 were used for the analysis of the investigated data.

## 4. Results

### 4.1. Descriptive Statistics of Respondents

The study distributed the survey to foreigners in potential markets except for China, the United States, Japan, Russia, and Kazakhstan, which are already the main customers of Korean medical tourism. The analysis results are as shown in Table 2. The number of male respondents was 118 (59.0%), and female was 82 (41.0%), with 35 from India (17.5%), 46 from Nepal (23.0%), 34 from Bangladesh (17.0%), 17 from Vietnam (8.5%), 45 from Mongolia (22.5%), and 23 from other nationalities (11.5%) such as Uzbekistan, Pakistan, Sweden, and New Zealand. There were three students who graduated from high school (1.5%), 94 university students (47.0%), 71 university graduates (35.5%), and 32 respondents above master’s degrees (16%). The expression of age is different in Korea and other countries, so the year of birth was surveyed and 92 respondents (46.0%) were born between 1993 and 1997.

### 4.2. Composite Reliability and Convergent/Discriminant Validity Testing

To verify the theoretical model, the results of confirmatory factor analysis (CFA) for each potential factor are shown in Table 3. Structural equation modeling (SEM) with a maximum likelihood method was used to test the relationships among constructs, following the two-step approach in which the measurement model was first confirmed. The second step is to test the structural model. The internal consistency and convergent validity of each construct were assessed. Cronbach’s alpha indicated adequate internal consistency of multiple indicators for each construct. Convergent validity was confirmed. All the standardized factor loadings on their underlying constructs; they were significant at the 0.001 level.

Cronbach’s coefficient alphas were again computed to obtain internal consistency estimates of reliability for the six constructs. The results showed that all six constructs met the minimum Cronbach’s coefficient reliability of 0.70, which indicated satisfactory internal consistency of each construct. A CFA was undertaken to assess the overall fit of the measurement model and to establish the convergent and discriminant validity of the constructs. The “goodness-of-fit” of the measurement model, as suggested by the fit indices, did not fit the data well. Therefore, based on the modification indices, a number of correlations between the errors of the variables of the same factor were added to the model. This modification did not violate the theoretical assumptions of the model because all correlations were within the same factor. After the modifications, the model had a reasonable fit to the data.

### 4.3. Correlations Analysis

To assess convergent validity, all factor loadings on their underlying constructs were evaluated. As shown in Table 3, except Disease Experience with Severity, Benefit and Visiting Intention, and Barrier with Visiting Intention, the other factor loadings for latent constructs were significant, suggesting convergent validity [38]. Moreover, the average variance extracted (AVE) of all constructs exceeded the minimum criterion of 0.538, indicating that a large portion of the variance was explained by the constructs. The AVEs were greater than the squared correlations between pairs of constructs, suggesting discriminant validity. The six-factor confirmatory measurement model demonstrated the soundness of its measurement properties. In summary, the assessment of the measurement model showed good evidence of reliability and validity for the operationalization of the latent constructs. Details of the properties of the measurements between study constructs are shown in Table 4.

### 4.4. Results of Hypothesis Test

**Figure 3:**
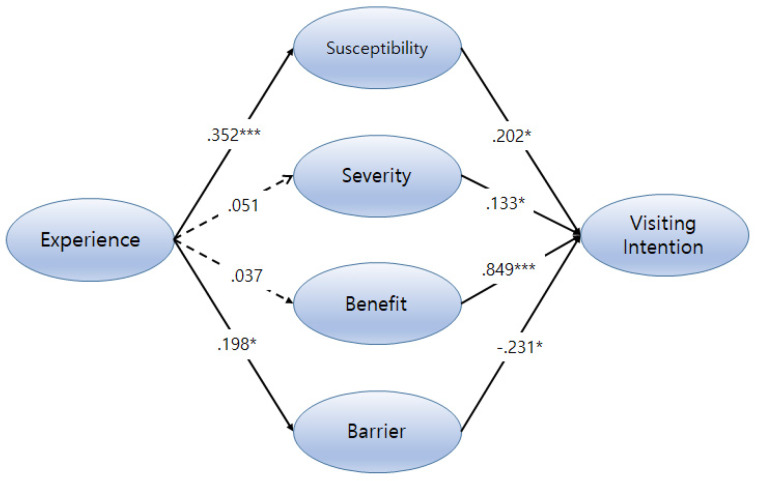
The results of testing the hypothesized model. (* *p* < 0.05 and *** *p* < 0.005).

The maximum likelihood estimation in AMOS 20.0 (IBM Corporation, Chicago, IL, USA) was used in the current study. SEM is well-suited for this type of analysis because it allows researchers to test models consisting of multiple outcomes and allows for the inclusion of variables that have potentially high correlations, such as Susceptibility, Severity, Benefit, and Barrier. This study was mainly designed to measure the impact of modified Health Beliefs on Visiting Intention to Korean medical tourism in two hypothesized ways: (a) via a direct relationship between Disease Experience and modified Health Beliefs, and (b) via a direct relationship through Health Beliefs and Visiting Intention. As specified in the analysis plan, a multivariate analysis was conducted to test these proposed relationships. The results of testing the hypothesized model are shown in Figure 3.

Hypothesis 1 proposed medical tourists with Disease Experience will have high health beliefs, which are Susceptibility (β = 0.352, *p* < 0.001) and Barrier (β = 0.198, *p* < 0.05) while Disease Experience was not significantly related in a positive way to their Severity (β = 0.352, *p* = 0.507) nor Benefit (β = 0.037, *p* = 0.621). Therefore, Hypotheses 1-2 and 1-3 were not supported by the path analysis. Hypothesis 2 proposed relationships between Health Beliefs and Visiting Intention. All Health Beliefs (Susceptibility, Severity, Benefit, and Barrier) had significant relationships with Visiting Intention. Benefit from Korean medical tourism had the most significant impact on Visiting Intention (β = 0.849, *p* < 0.001). Path analysis found that reversed Barrier scores had a negative impact on Visiting Intention (β = −0.231, *p* < 0.05). This result of analysis for Hypothesis 2-2 showed that foreigners who believe they have a low level of Barrier are willing to have a positive intention to be involved in Korean medical tourism. A summary of the outcomes for the hypotheses is presented in Table 5.

## 5. Discussion

This study seeks to find out the Visiting Intention to Korea to use medical services for cancer treatment based on HBM. In summary, these results showed that a high level of Benefit had a significant effect, and Barrier had a negative impact on Visiting Intention to Korean medical tourism. This fact would lead to positive behavioral intentions to experience advanced medical services in Korea with tourism. The results of the study were inconsistent with prior studies. Huang, Dai, and Xu [39] explored the relationships underlying travelers’ health beliefs, attitudes, self-efficacy, preventive behaviors, and traveling satisfaction for Tibet tourists. The perceived Severity was not supported as preventative behavior, and perceived Susceptibility and perceived Benefit had a significant impact on the preventative behavior in this research. One thing in common with this study is that Benefit had a positive effect on behavior. In addition, Han and Hyun [40] developed a model explaining international medical travelers’ intention formation by considering the impact of quality, satisfaction, trust, and price reasonableness. A field survey was conducted at medical clinics. Findings from the structural analysis indicate a good fit for the proposed model; perceived quality, satisfaction, and trust in the staff and clinic have significant associations affecting intentions to revisit clinics and the destination country, and satisfaction and trust acted as significant mediators. Compared with the result of this study, the perceived Benefit could be a similar concept to the impact of quality and trust of clinics. Therefore, it is considered to be the result of supporting prior study.

The relationship between Susceptibility and Visiting Intention is controversial because of differences in the concept of components and inconsistencies in definitions. Many studies defined a positive (+) relationship between Susceptibility and Visiting Intention [34,36,41,42,43,44]. On the other hand, many other studies have defined this relationship as negative [39,45]. The relationship between Susceptibility and Visiting Intention has been shown significantly in this study, which is judged to be a result of support for many prior studies claiming that the relationship between perceived Susceptibility and behavior is positive.

The results clearly showed that, as hypothesized, two direct measures were significant predictors of Disease Experience. Both Susceptibility and Barrier had a significant effect on Visiting Intention and were a valid measurement to use to determine Visiting Intention. Additionally, it was determined that Severity and Benefit were not significant predictors of Disease Experience, but those were significant factors to Visiting Intention. Susceptibility, Severity, and Benefit have been shown to have a positive impact on Visiting Intention. Relative influence is the sequence of Benefits, Susceptibility, and Severity. The results showed that the benefits of foreigners using Korean medical tourism are the most important factor in their intention to visit Korea. If they have cancer, they want to visit Korea for treatment because of their Severity, which is a subjective assessment of the results, and their Susceptibility, which is a personal assessment of the possibility of cancer. Finally, this study helped to validate the use of the Health Belief Model in medical tourism.

However, Barrier has been shown to have a negative impact on the Visiting Intention. This indicates that if they have cancer, the fewer barriers to visiting Korea and receiving medical services will mean the more willing they are to visit Korea. Therefore, it is desirable for the Korean medical tourism marketers to actively promote medical services through information brochures, such as international exhibitions on medical services and exhibitions of medical equipment. In particular, it is necessary to communicate with Korean medical staff while increasing the accessibility of Korean medical services by actively utilizing Internet portal sites. Thus, it is necessary to lower the level of disability by providing clearer information on what foreigners believe will be an obstacle to the use of Korean medical services.

Despite the significant findings, this study has the following limitations. First, it is acknowledged that this study has limitations in generalizing the areas of respondents because they were designated only as countries other than Korea. Therefore, it is deemed that the areas subject to the survey should be clarified in future research to generalize the result. Second, based on the results of this study, it is also considered that it will be a good study to use cultural differences to identify the relationship between perception and intent to visit. Generally, the relationship between people’s perceptions and behaviors varies from culture to culture [46]. Therefore, it is believed that it will be meaningful to examine people in other cultures as respondents and identify cultural differences and intentions of visiting Korean for medical tourism. Moreover, it would be more meaningful to study the concept of expanded medical tourism by comparing the perception of medical tourism in other countries that have already developed through medical tourism with the perception of medical tourism in medical tourism in Korea.

## 6. Conclusions

HBM, as a highly well-known structure, has been widely used in research and it predicts Visiting Intention to receive some benefits to cure diseases, such as cancer, besides plastic surgery. The results of the current study provide empirical evidence of medical tourists’ visiting intention. It shows that the Disease Experience leads to perceived Susceptibility and Barrier factors, and influences Visiting Intention. The other two factors (Severity and Benefit) increase Visiting Intention to Korean medical tourism. This result shows that a person with a history of painful experiences or a family history of cancer is highly perceptible to the risk that he or she may have cancer and has a barrier to cure the cancer. In addition, the severity and susceptibility of cancer and the benefits of using Korean medical services increase the willingness to visit Korea. Finally, this study helps to validate the use of the Health Belief Model in medical tourism.

## Figures and Tables

**Table 1:** Composition of questionnaire.

Factors	Abbreviation	Items Measurement	Sources
Disease Experience	EX 1	I have a family history of cancer.	Kim, Myoung and Kim [35]
	EX 2	I have many painful experiences.	
Susceptibility	SU 1	I think cancer can cause unbearable pain.	Kim, Myoung and Kim [35]
	SU 2	If I get cancer, my life will be destroyed.	Kim, Ahn and No [36]
	SU 3	If I get cancer, I will not get proper treatment in my country.	Kim [37]
Severity	SE 1	I was sick and had some inconvenience in my daily life.	Kim, Myoung and Kim [35]
	SE 2	I often suffered very much when I was sick.	Kim, Ahn and No [36]
	SE 3	I have a high chance of getting cancer.	Kim [37]
Benefit	BE 1	Korean medical services will help cure cancer.	Kim, Myoung and Kim [35] Kim, Ahn and No [36]
	BE 2	Korean medical services will be able to minimize the recurrence of cancer.	
	BE 3	Korean medical services help prevent further diseases.	
	BE 4	Korean medical services to treat cancer will reduce stress.	
Barrier	BA 1	Korean medical services for cancer treatment are an obstacle to daily life.	Kim, Myoung and Kim [35] Kim, Ahn and No [36] Kim [37]
	BA 2	Korean medical services for cancer treatment will cost a lot.	
	BA 3	Korean medical services for cancer treatment are time-consuming.	
	BA 4	Korean medical service for cancer treatment is a complex task. (e.g., visa, accommodation, and language)	
Visiting Intention	VI 1	I will consider using Korean medical services if I get cancer.	Kim, Myoung and Kim [35] Kim [37]
	VI 2	I am willing to use Korean medical services if I get cancer.	
	VI 3	I want to use Korean medical service even if it costs a lot.	
	VI 4	I will recommend using Korean medical services to treat cancer to others.	

**Table 2:** General characteristics of the respondents.

	Characteristic	Frequency (N)	Percent (%)
Gender	Male	118	59.0
	Female	82	41.0
Nationality	India	35	17.5
	Nepal	46	23.0
	Bangladesh	34	17.0
	Vietnam	17	8.5
	Mongol	45	22.5
	Others	23	11.5
Education	Less than high school degree	3	1.5
	University student	94	47.0
	Bachelor’s degree	71	35.5
	Above graduate degree	32	16.0
Birth year	2002~1998	56	28.0
	1993~1997	92	46.0
	1989~1992	19	9.5
	~1988	33	16.5
Total		200	100

**Table 3:** Results of composite reliability and convergent/discriminant validity testing.

Factors	Variable	Intensive Validity				Concept Reliability		Cronbach α
		Estimate		SE	*t*-Value	CR	AVE	
		B	β					
Disease Experience	EX 1	1.000	0.940			0.993	0.986	0.778
	EX 2	3.0570	0.999	0.093	38.333			
Susceptibility	SU 1	1.000	0.768			0.651	0.847	0.736
	SU 2	1.845	0.966	0.317	5.811			
	SU 3	1.661	0.850	0.297	5.584			
Severity	SE 1	1.000	0.868			0.686	0.867	0.704
	SE 2	1.256	0.951	0.210	5.972			
	SE 3	0.603	0.788	0.172	5.068			
Benefit	BE 1	1.000	0.817			0.706	0.905	0.891
	BE 2	1.107	0.879	0.088	12.538			
	BE 3	1.228	0.951	0.089	13.761			
	BE 4	1.072	0.835	0.091	11.741			
Barrier	BA 1	1.000	0.910			0.660	0.886	0.750
	BA 2	0.708	0.878	0.138	5.117			
	BA 3	0.847	0.903	0.148	5.725			
	BA 4	0.909	0.856	0.166	5.468			
Visiting Intention	VI 1	1.000	0.970			0.657	0.884	0.884
	VI 2	1.042	0.982	0.060	17.506			
	VI 3	0.877	0.871	0.081	10.860			
	VI 4	0.898	0.928	0.059	15.126			

**Table 4:** Convergent/discriminant validity.

	Disease Experience	Susceptibility	Severity	Benefit	Barrier
Susceptibility	0.084 *** (0.007)				
Severity	0.019 (0.000)	0.241 *** (0.058)			
Benefit	0.007 (0.000)	0.115 * (0.013)	0.233 ** (0.054)		
Barrier	0.062 * (0.004)	0.226 *** (0.051)	0.260 ** (0.068)	0.194 ** (0.038)	
Visiting Intention	0.047 (0.002)	0.187 ** (0.034)	0.346 *** (0.120)	0.729 *** (0.531)	0.124 (0.015)

* *p* < 0.05, ** *p* < 0.01 and *** *p* < 0.001.

**Table 5:** Structural equation results.

	Hypothesized Relationship			Estimate		S.E.	C.R.	Result
				B	β			
H1-1	Disease Experience	→	Susceptibility	0.457	0.352	0.122	3.729 ***	Supported
H1-2	Disease Experience	→	Severity	0.116	0.051	0.174	0.664	Reject
H1-3	Disease Experience	→	Benefit	0.176	0.037	0.154	0.495	Reject
H1-4	Disease Experience	→	Barrier	0.365	0.198	0.162	20.252 *	Supported
H2-1	Susceptibility	→	Visiting Intention	0.393	0.202	0.168	2.335 *	Supported
H2-2	Severity	→	Visiting Intention	0.149	0.133	0.073	2.046 *	Supported
H2-1	Benefit	→	Visiting Intention	1.023	0.849	0.100	10.262 ***	Supported
H2-2	Barrier	→	Visiting Intention	−0.316	−0.231	0.124	−2.547 *	Supported

*X*^2^ = 351.145, (DF = 152, *p* < 0.000), *X*^2^/DF = 2.310, CFI = 0.906, NFI = 0.848, IFI = 0.907, TLI = 0.882, RMSEA = 0.081. * *p* < 0.05 and *** *p* < 0.005.

## References

[1] Korea Tourism Organization Korean Medical Tourism Marketing. The Present and Future of Korean Medical Tourism. https://kto.visitkorea.or.kr/viewer/view.kto?id=47773&type=bd.

[2] Byun J.W., Kim Y.K. Current Status and Strategy of Promotional Marketing for Medical Tourism. https://kto.visitkorea.or.kr/kor/biz/marketing/medical/data.kto.

[3] Song M.K., Kim S.H. (2016). A study on the difference of medical tourism storytelling attributes by the medical tourists" behavior characteristic and demographic characteristic-Focused on Chinese and Japanese medical tourist in Korea. Int. J. Tour. Hosp. Res..

[4] Beladi H., Chao C.C., Ee M.S., Hollas D. (2019). Does medical tourism promote economic growth? A cross-country analysis. J. Travel Res..

[5] Visit Medical Korea Strengths of the Korean Medical System. https://kto.visitkorea.or.kr/viewer/view.kto?id=47773&type=bd.

[6] Rhee N.H., Cho S.W., Jin S.N. (2016). A study on causal relationship between selection attributes of Russian medical tourists to Korea and their intention of visiting Korea. J. Tour. Leisure Res..

[7] Kim S., Choi S. (2015). The medical professionalism of Korean physicians: Present and future. BMC Med. Ethics.

[8] Cho K.T., Kim S.M. (2003). Selecting medical devices and materials for development in Korea: The analytic hierarchy process approach. Int. J. Health Plan. Manag..

[9] Eom T., Yu J., Han H. (2019). Medical tourism in Korea–recent phenomena, emerging markets, potential threats, and challenge factors: A review. Asia Pac. J. Tour. Res..

[10] Jang S.H., Lee E.J., Lim J.A., Vu T., Taylor V.M., Ko L.K. (2019). The role of medical tourism in cancer screening among Korean immigrant women. Health Behav. Policy Rev..

[11] Fetscherin M., Stephano R.M. (2016). The medical tourism index: Scale development and validation. Tour. Manag..

[12] Saragih H.S., Jonathan P. (2019). Views of Indonesian consumer towards medical tourism experience in Malaysia. J. Asia Bus. Stud..

[13] Hallem Y., Barth I. (2015). Understanding the role of Internet in explaining the medical-tourist behavior: A conceptual model. Manag. Avenir Sante.

[14] Kim S., Arcodia C., Kim I. (2019). Critical Success Factors of Medical Tourism: The Case of South Korea. Int. J. Environ. Res. Public Health.

[15] Kim J.H., Cho J.H. (2019). Investigation of effects of individuals’ social viewing of fine dust information obtained through social media on behavioral intentions of disease prevention: Application of health beliefs model. Korea Broadcast. Tellecommun. Stud..

[16] Hochbaum G.M. (1958). Public Participation in Medical Screening Programs: A Socio-Psychological Study.

[17] Rosenstock I.M. (1974). Historical origins of the health belief model. Health Educ. Quart..

[18] Rosenstock I.M., Strecher V.J., Becker M.H. (1988). Social learning theory and the health belief model. Health Educ. Quart..

[19] Ban H.J., Kim H.S. (2019). Understanding customer experience and satisfaction through airline passengers’ online review. Sustainability.

[20] Koo K.Y., Kim M.S. (2013). The study of medical tourism plastic cosmetice surgery satisfaction/dis-satisfaction: Chinese tourist. J. Tour. Leisure Res..

[21] Yang E.J., Cho M.H. (2015). A study on the influence factors of behavior intention for Chinese cosmetic surgery tourism: Focusing on extended model of goal-directed behavior. J. Tour. Sci..

[22] Joo Y.J. (2017). The effect of service quality during medical tour of Chinese on the satisfaction and revisit intention. Int. J. Tour. Manag. Sci..

[23] Chang Y.H., Shin D.S. (2018). Impacts of Chinese tourists’ beauty therapy and plastic surgery motivation on satisfaction. J. Tour. Manag. Res..

[24] Jeong Y.R. (2019). The effects of Halyu-star image and medical tourism service quality on cosmetic surgery attitude and behavioral intention: The case of latent Chinese female. J. Tour. Leisure Res..

[25] OECD Indicators (2015). Health at a Glance. https://read.oecd-ilibrary.org/social-issues-migration-health/health-at-a-glance-2015_health_glance-2015-en#page1.

[26] Korea Tourism Organization Medical Tourism. https://kto.visitkorea.or.kr/kor/biz/marketing/medical.kto.

[27] Medical Kore Statistical Analysis Report on Attraction of Foreign Patients. https://www.medicalkorea.or.kr/notice/infoView.do.

[28] Kim Y.J., Kim J. (2018). Effects of Expected Medical Service and Country Image on Medical Tourism Intention. Inter. Bus. Rev..

[29] Janz N.K., Becker M.H. (1984). The health belief model: A decade later. Health Educ. Quart..

[30] Larsen P.D. (2002). Chronic Illness: Impact and Interventions.

[31] Ajzen I. (2002). Perceived behavioral control, self-efficacy, locus of control, and the theory of planned behavior 1. J. Appl. Soc. Psychol..

[32] Chew F., Palmer S., Slonska Z., Subbiah K. (2002). Enhancing health knowledge, health beliefs, and health behavior in Poland through a health promoting television program series. J. Health Commun..

[33] Chien P.M., Sharifpour M., Ritchie B.W., Watson B. (2017). Travelers’ health risk perceptions and preventative behavior: A psychological approach. J. Travel Res..

[34] Mehraban S.S.Z., Namdar A., Naghizadeh M.M. (2018). Assessment of preventive behavior for cervical cancer with the health belief model. Asian Pac. J. Cancer P.

[35] Kim Y.K., Myoung H., Kim Y.B. (2012). The effect of Chinese perception on buying intention toward dental treatment of South Korea: Shanghai residents based on HBM(Health Belief Model). Korean J. Hotel Tour..

[36] Kim H.S., Ahn J., No J.K. (2012). Applying the Health Belief Model to college students’ health behavior. Nutr. Res. Pract..

[37] Kim (2019). A study of the effects on perceived risk on overall risk and intention to participate in medical tourism abroad: Focused on US potential medical tourists. Tour. Res..

[38] Gerbing D.W., Anderson J.C. (1988). An updated paradigm for scale development incorporating unidimensionality and its assessment. J. Mark. Res..

[39] Huang X., Dai S., Xu H. (2020). Predicting tourists’ health risk preventative behaviour and travelling satisfaction in Tibet: Combining the theory of planned behaviour and health belief model. Tour. Manag. Perspect..

[40] Han H., Hyun S.S. (2015). Customer retention in the medical tourism industry: Impact of quality, satisfaction, trust, and price reasonableness. Tour. Manag..

[41] Li C., Unger J.B., Schuster D., Rohrbach L.A., Howard-Pitney B., Norman G. (2003). Youths’ exposure to environmental tobacco smoke (ETS): Associations with health beliefs and social pressure. Addict. Behav..

[42] Umeh K., Rogan-Gibson J. (2001). Perceptions of threat, benefits, and barriers in breast self-examination amongst young asymptomatic women. Br. J. Heal. Psychol..

[43] Lee M.S. (2001). A Study on the Relationship between Adolescent Misconducts and Harmful Environment Based on Health Belief Model. Korean J. Heal. Educ. Pr..

[44] Larki A., Tahmasebi R., Reisi M. (2018). Factors predicting self-care behaviors among low health literacy hypertensive patients based on health belief model in Bushehr District, South of Iran. Inter. J. Hypertens..

[45] Wiebe J.S., Christensen A.J. (1997). Health beliefs, personality, and adherence in hemodialysis patients: An interactional perspective. Ann. Behav. Med..

[46] Sheldon P., Rauschnabel P.A., Antony M.G., Car S. (2017). A cross-cultural comparison of Croatian and American social network sites: Exploring cultural differences in motives for Instagram use. Comp. Hum. Behav..

